# HLA-A*03, the hemochromatosis ancestral haplotype, and phenotypes of referred hemochromatosis probands with *HFE* p.C282Y homozygosity

**DOI:** 10.1186/s41065-022-00237-w

**Published:** 2022-06-06

**Authors:** James C. Barton, J. Clayborn Barton, Ronald T. Acton

**Affiliations:** 1 Southern Iron Disorders Center, Birmingham, AL USA; 2 Department of Medicine, University of Alabama at Birmingham, Birmingham, AL USA; 3 Department of Microbiology, University of Alabama at Birmingham, Birmingham, AL USA

**Keywords:** Ancestral haplotype, APRI, FIB-4, Haplotype, Iron overload

## Abstract

**Background:**

Human leukocyte antigen (HLA)-A*03, hemochromatosis ancestral haplotype marker, was associated with greater iron overload in hemochromatosis cohorts reported before discovery of the *HFE* gene. We sought to learn whether an A*03-linked locus influences phenotypes in referred *HFE* p.C282Y homozygotes.

**Methods:**

We tabulated these phenotypes in probands with p.C282Y homozygosity: age, transferrin saturation (TS), serum ferritin (SF), conditions related to iron overload, fibrosis-four variables (FIB-4) index and aspartate aminotransferase-to-platelet ratio index (APRI) predictors of severe hepatic fibrosis, and iron removed to achieve depletion (QFe/age). We analyzed phenotypes of men and women separately across three A*03 subgroups.

**Results:**

There were 104 men (57.8%) and 76 women (42.2%). Mean age (SD) was 49 ± 13 y. Mean TS was 79 ± 17%. Median SF (range) was 715 µg/L (28, 6103). Related conditions included: hemochromatosis arthropathy (21.7%); type 2 diabetes (18.9%); hypogonadotropic hypogonadism (5.8% of men); cardiomyopathy (0%); and cirrhosis (10.0%). Median QFe/age was 61 mg/y (0, 714). A*03 homozygosity, heterozygosity, and no A*03 occurred in 37 (20.6%), 104 (57.8%), and 39 probands (21.7%), respectively. In men, mean TS and median SF were significantly higher in A*03 homozygotes than heterozygotes but not A*03-negative probands. In men, median APRI was significantly lower in A*03 heterozygotes than homozygotes and A*03-negative probands. No other phenotypes, including QFe/age, differed significantly across A*03 subgroups in either men or women.

**Conclusions:**

Our results suggest that an A*03-linked locus does not influence phenotypes in referred *HFE* p.C282Y homozygotes. It is unlikely that heritable factors that modify phenotypes of p.C282Y homozygotes are linked to the hemochromatosis ancestral haplotype.

## Introduction

Hemochromatosis in whites of western European descent is characterized by elevated transferrin saturation (TS) and serum ferritin (SF) levels and increased risk to develop iron overload and related conditions, including hemochromatosis arthropathy, diabetes mellitus, hypogonadotropic hypogonadism, cardiomyopathy, and hepatic cirrhosis [1]. Severe iron overload due to hemochromatosis occurs predominantly in men [1]. Other heritable and environmental factors modify iron loading in adults with hemochromatosis [1, 2]. Regardless, phenotype variability in hemochromatosis [1, 3] remains largely unexplained.

Human leukocyte antigen (HLA)-A*03 was among the early A series of HLA alleles discovered by Bodmer et al. in 1966 [4] and recognized by the World Health Organization Leukocyte Nomenclature Committee in 1968 [5]. Antisera to A*03 were standardized through subsequent International Histocompatibility Workshops. A*03, a cell-surface protein of 365 amino acids involved in short polypeptide presentation to T-lymphocytes, is encoded by *HLA-A* (chromosome 6p22.1) [6]. In whites in western European and derivative countries, A*03:01 predominates and A*03:02 is less common [7, 8]. Both alleles react with anti-A*03 serotyping antibodies.

In 1975, Simon and colleagues reported that a factor associated with hemochromatosis in French patients was linked to HLA-A*03 [9, 10]. In 1987, they reported that HLA-A*03, B*07, A*03, B*14, and other A, B haplotypes were in linkage disequilibrium with presumed hemochromatosis alleles, although A*03 alone was independently associated with hemochromatosis [11]. Simon and colleagues hypothesized that the presumed hemochromatosis allele was "a rare if not unique event that produced an ancestral HLA marking that was subsequently modified by recombinations and geographical scattering due to migrations" [11].

In a 1995 study of Australian hemochromatosis patients and their families, Jazwinska and colleagues defined the chromosome 6p hemochromatosis ancestral haplotype (AH) as D6S248(5)-D6S265(1)-HLA-A*03-HLA-F*02-D6S105(8) [12]. Among these alleles, only A*03 was an independent surrogate marker for the AH [12], consistent with the previous report of Simon and colleagues [11]. Australian hemochromatosis patients with two A*03 alleles had greater mean TS and mean hepatic iron indices than hemochromatosis patients with one or no A*03 [13]. In a 1996 study of Alabama hemochromatosis probands, mean age-adjusted units of blood removed to deplete body iron stores was significantly greater in probands with A*03 or D6S105(8) homozygosity. Intermediate values of mean units of blood removed were observed in both A*03 and D6S105(8) heterozygotes [14]. In a 1996 study of Italian men with hemochromatosis, greater amounts of iron were removed by phlebotomy, with or without adjustment for age at diagnosis, in A*03 homozygotes and heterozygotes than those without A*03 [15]. Taken together, these studies [13–15] supported the postulate that a locus linked to A*03 influences hemochromatosis phenotypes [16].

In 1996, Feder and colleagues discovered the *HFE* gene (homeostatic iron regulator, chromosome 6p22.2) and two common *HFE* missense alleles in patients with hemochromatosis [17]. Among 178 patients, 83% were *HFE* p.C282Y homozygotes, 16% were p.C282Y/p.H63D compound heterozygotes, and one was a p.H63D homozygote [17]. This discovery confirmed that there is a predominant hemochromatosis allele (p.C282Y) and that combinations of *HFE* alleles (p.C282Y, p.H63D) on different haplotypes account for hemochromatosis phenotypes. Subsequently, many other pathogenic *HFE* alleles have been identified in persons with hemochromatosis [18], although it remains unknown whether a locus linked to A*03 influences hemochromatosis phenotypes in p.C282Y homozygotes [16].

To learn more, we performed a retrospective study of 180 consecutive, referred Alabama hemochromatosis probands with *HFE* p.C282Y homozygosity (104 men, 76 women) who underwent HLA-A typing and achieved iron depletion with phlebotomy. We sought to determine whether A*03 homozygosity, A*03 heterozygosity, or no A*03 is significantly associated with these phenotypes: age, TS, SF, conditions related to iron overload (hemochromatosis arthropathy, diabetes, hypogonadotropic hypogonadism, and cirrhosis) at diagnosis, and age-adjusted quantity of iron removed to achieve depletion (QFe/y, mg/y). We discuss our results in the context of the three studies of patients with hemochromatosis with and without A*03 published before the discovery of *HFE* [13–15]. We review evidence for the existence of a locus linked to A*03 that influences phenotypes in p.C282Y homozygotes.

## Methods

### Subjects included

We compiled observations on 180 consecutive self-identified non-Hispanic white adults ≥ 18 y referred to an Alabama tertiary center (1990–2020) for evaluation of hemochromatosis who a) were *HFE* p.C282Y homozygotes, b) underwent HLA-A typing, c) achieved iron depletion with phlebotomy at this center, and d) were the first in their respective families to be diagnosed to have hemochromatosis (probands). Medical histories were taken from probands and records of referring physicians. All probands underwent physical examination, laboratory testing, imaging procedures, and evaluation of liver conditions, as indicated, before therapeutic phlebotomy was initiated.

### Subjects excluded

We excluded *HFE* p.C282Y homozygotes who a) were family members of the present probands, b) presented with iron deficiency (defined as both TS < 10% and SF < 15 µg/L), c) were treated with phlebotomy or iron chelation before referral, or d) had incomplete evaluations. We also excluded probands diagnosed to have hemochromatosis because they participated in the Hemochromatosis and Iron Overload Screening (HEIRS) Study [19, 20] or other screening programs.

### Laboratory methods

TS, SF, and serum levels of total testosterone, estradiol, luteinizing hormone (LH), follicle-stimulating hormone (FSH), alanine aminotransferase (ALT), and aspartate aminotransferase (AST) were measured using morning specimens obtained without regard to fasting and standard clinical laboratory methods (Laboratory Corporation of America, Burlington, NC, USA). Platelet counts were measured using automated hematology analyzers (Cell-Dyn® Models 610 and 1700, Abbott Laboratories, Chicago, IL, USA).

*HFE* allele analyses were performed using standard clinical laboratory methods (Laboratory Corporation of America, Burlington, NC, USA) as previously described [16]. HLA-A phenotypes were determined using the microdroplet lymphocytotoxicity assay [14], HLA Class I SSPARMS-PCR typing kits [21, 22], or sequence-based typing (Laboratory Corporation of America, Burlington, NC, USA). Herein, we use the term A*03 whether A*03 was identified by serotyping or DNA-based typing.

### Hemochromatosis ancestral haplotype

We defined HLA-A*03 as the marker of the AH, consistent with previous reports [11, 13–15]. We defined A*03 positivity as A*03 homozygosity or heterozygosity. We defined A*03 allele frequency as the quotient of total A*03 alleles by total HLA-A alleles.

### Conditions related to iron overload

We defined conditions related to iron overload as hemochromatosis arthropathy, diabetes, hypogonadotropic hypogonadism, cardiomyopathy, and cirrhosis.

### Hemochromatosis arthropathy

We defined hemochromatosis hand arthropathy as the combination of tenderness, pain, swelling, or effusion of the second and third metacarpophalangeal joints [3, 23] and radiological features consistent with those observed in hemochromatosis [1] in the absence of other cause(s). We defined hemochromatosis non-hand arthropathy as physical examination and radiological abnormalities in shoulders, elbows, hips, knees, and ankles typical of hemochromatosis [1] in the absence of other cause(s). We classified probands who had either hemochromatosis hand arthropathy, hemochromatosis non-hand arthropathy, or both as having hemochromatosis arthropathy. We did not classify autoimmune or neuropathic arthropathy as hemochromatosis arthropathy. We did not evaluate observations on spine or other arthropathy.

### Diabetes

Diabetes was diagnosed by referring physicians and classified according to criteria of the American Diabetes Association [24].

### Hypogonadotropic hypogonadism

Serum levels of total testosterone, LH, and FSH were measured at diagnosis of hemochromatosis in men who reported loss of libido or erectile dysfunction due to undefined cause(s) and did not take supplemental androgen(s). Serum levels of estradiol, LH, and FSH were measured at diagnosis of hemochromatosis in women who reported premature cessation of menses not due to menopause or other defined cause(s) and did not take hormone replacement therapy. Hypogonadotropic hypogonadism was defined as the combination of subnormal serum total testosterone levels < 241 ng/dL (< 8.4 nmol/L) (men) or serum estradiol levels < 30 pg/mL (<110 pmol/L) (women) and serum LH and FSH levels that did not exceed the upper reference limits (9.3 IU/L and 18.1 IU/L, respectively), in the absence of other cause(s) [25].

### Cardiomyopathy

Hemochromatosis cardiomyopathy was defined as dilated cardiomyopathy with low left ventricular ejection fraction and decreased fractional shortening, with or without complete atrioventricular block or atrial and ventricular tachyarrhythmias, due to cardiac siderosis demonstrated by endomyocardial biopsy or MRI scanning [1, 26].

### Cirrhosis

We recommended that probands undergo percutaneous liver biopsy (or not) in accordance with hemochromatosis diagnosis and management guidelines of the American Association for the Study of Liver Diseases [27]. Iron staining in liver specimens was graded 0–4 + as previously described [28]. Hepatic iron was measured using atomic absorption spectrometry. Pathologists interpreted liver specimens and defined cirrhosis as the histological occurrence of regenerating nodules of hepatocytes surrounded by bands of fibrous connective tissue [29]. In some probands, the presence or absence of cirrhosis was ascertained using imaging techniques (CT scanning, abdominal ultrasonography, or elastography) [30].

### FIB-4 and APRI

We computed the fibrosis-four variables (FIB-4) index [31] and the AST-to-platelet ratio index (APRI) [32] in all probands. Both are moderately accurate non-invasive indices originally derived to identify patients with hepatitis virus C infection with increased cirrhosis risk. Subsequently, FIB-4 and APRI have been studied in *HFE* p.C282Y homozygotes [33].

Threshold (or greater) values of each index are associated with increased risk for advanced hepatic fibrosis (stage F3, severe fibrosis with architectural distortion or stage F4, cirrhosis with architectural distortion) [34]. A FIB-4 index > 1.10 identified *HFE* p.C282Y homozygotes with advanced fibrosis with 80% sensitivity, 80% specificity, and 81% accuracy [33]. APRI values > 0.44 identified p.C282Y homozygotes with advanced hepatic fibrosis with 79% sensitivity, 79% specificity, and 81% accuracy [33].

### Iron removed by phlebotomy (QFe)

Iron depletion therapy, defined as the periodic removal of blood to eliminate storage iron, was performed in probands with elevated SF at diagnosis as previously defined (> 300 µg/L, men; SF > 200 µg/L, women) [19]. Iron depletion therapy was complete when SF was ≤ 20 μg/L [35]. QFe was estimated to be 200 mg Fe per unit of blood (450–500 mL) [35]. Herein, we defined QFe to be zero in men with SF ≤ 300 µg/L at diagnosis and women with SF ≤ 200 µg/L at diagnosis and thus such probands did not undergo therapeutic phlebotomy. We defined QFe/age at diagnosis (mg/y) as a measure of iron overload severity.

### Statistical analyses

The sample size of 180 probands represented the entirety of referred Alabama probands with hemochromatosis and *HFE* p.C282Y homozygosity diagnosed during the study interval (1990–2020) whose medical records met all stipulations for inclusion in this study. The data we analyzed are available in an open-access database [36]. Age, TS, and SF at diagnosis and QFe/age data are displayed to the nearest integer. Evaluation of continuous data using d'Agostino’s and Shapiro–Wilk tests revealed that age and TS values were normally distributed. Results are displayed as mean ± 1 standard deviation (SD) and were compared with Student’s t tests or one-way analysis of variance (ANOVA) tests and post-hoc Scheffe’s tests, as appropriate. SF, QFe/age, FIB-4 index, and APRI data were not normally distributed. For APRI computations, we used AST 40 IU/L as the upper limit of normal. For some comparisons, we used these threshold values: FIB-4 > 1.10 and APRI > 0.44 [33]. Results are displayed as median (range) and were compared with either Mann–Whitney U tests or Kruskal–Wallis tests and post-hoc Dunn’s tests, as appropriate.

Proportions for two categories were compared using Fisher's exact test (two-tailed). Proportions for three or four categories were evaluated with Pearson’s Chi-square tests (3 × 2 or 4 × 2 tables) and compared post-hoc using 2 × 2 Fisher’s exact tests (two-tailed), as appropriate. In one analysis, we compared HLA-A*03 positivity and allele frequency in men and women, respectively, grouped by the highest quartile of QFe/age with those grouped by the lowest three quartiles of QFe/age. Values of p < 0.05 were defined as significant. Analyses were performed with Excel 2000® (Microsoft Corp., Redmond, WA, USA), GB-Stat® (2003, Dynamic Microsystems, Inc., Silver Spring, MD, USA), and GraphPad Prism 8® (2018, GraphPad Software, San Diego, CA, USA).

## Results

### Phenotypes of 180 probands

There were 104 men (57.8%) and 76 women (42.2%). Mean age (SD) at diagnosis of hemochromatosis was 49 ± 13 y. Mean TS was 79 ± 17%. Median SF (range) was 715 µg/L (28, 6103). SF was > 1000 µg/L (1001, 6103) in 65 probands (36.1%; 50 men, 15 women).

Hemochromatosis arthropathy was diagnosed in 39 probands (21.7%). Diabetes was diagnosed in 34 probands (18.9%), each of whom had type 2 diabetes. Hypogonadotropic hypogonadism was diagnosed in six men (6/104, 5.8%) and no women. No proband was diagnosed to have cardiomyopathy due to cardiac siderosis.

Liver specimens were obtained by biopsy in 20 of 65 probands with SF > 1000 µg/L (30.8%) and 41 of 115 probands with SF ≤ 1000 µg/dL (35.7%) (p = 0.6231). The other 45 probands with SF > 1000 µg/L were evaluated for cirrhosis with imaging studies [30]. Altogether, cirrhosis was diagnosed in 18 probands (18/180, 10.0%), 15 of whom (15/18, 83.3%) had SF > 1000 µg/L. In 17 probands with cirrhosis (17/18, 94.4%), the sole or predominant cause of cirrhosis was hepatic iron overload. The other proband with cirrhosis was a woman with SF 387 µg/L and hepatic sarcoidosis. No proband was discovered to have primary liver cancer at diagnosis of hemochromatosis.

Therapeutic phlebotomy to achieve iron depletion was indicated and performed in 178 probands (98.9%). Median QFe/age (range) was 61 mg/y (0, 714).

Homozygosity for A*03, heterozygosity for A*03, and no A*03 was detected in 37 probands (20.6%), 104 probands (57.8%), and 39 probands (21.7%), respectively.

### Phenotypes of 104 men with hemochromatosis

These results are displayed in Table 1. In men, mean TS and median SF were significantly higher in A*03 homozygotes than heterozygotes but not A*03-negative men. Prevalences of conditions related to iron overload and median QFe/age did not differ significantly across subgroups of men with A*03 homozygosity, A*03 heterozygosity, or no A*03.

**Table 1:** Phenotypes of 104 men with hemochromatosis and *HFE* p.C282Y homozygosity ^a^

Phenotype	HLA-A*03 homozygotes, % (*n* = 21)	HLA-A*03 heterozygotes (*n* = 66)	No HLA-A*03, % (*n* = 17)	Value of *p*
Mean age, y ± 1 SD	46 ± 12	48 ± 15	47 ± 12	0.6251
Mean TS, % ± 1 SD	88 ± 16	80 ± 16	81 ± 13	< 0.0001 ^b^
Median SF, µg/L (range)	1362 (389, 6103)	867 (28, 5613)	1112 (285, 5001)	0.0252 ^c^
Hemochromatosis arthropathy, % (n)	19.0 (4)	21.2 (14)	41.2 (7)	0.1911
Diabetes mellitus, % (n)	33.3 (7)	13.6 (9)	29.4 (5)	0.0860
Hypogonadotropic hypogonadism ^d^, % (n)	9.5 (2)	3.0 (2)	11.8 (2)	0.2754
Cardiomyopathy, % (n)	0	0	0	1.0000
Cirrhosis, % (n)	16.0 (5)	9.1 (6)	23.5 (4)	0.1248
Median FIB-4 index (range)	1.14 (0.37, 2.64)	1.12 (0.33, 6.16)	1.26 (0.65, 2.46)	0.2595
Median APRI (range)	0.46 (0.17, 5.31)	0.31 (0.13, 2.75)	0.48 (0.20, 1.28)	0.0162 ^e^
Median QFe/age, mg/y (range)	71 (14, 213)	61 (0, 714)	73 (8, 320)	0.6386

^a^ Abbreviations: *APRI*, aspartate aminotransferase-to-platelet ratio, *FIB-4* fibrosis-four variables index, *HLA* human leukocyte antigen, QFe/age is defined as the quotient of quantity of iron removed to achieve iron depletion by age at diagnosis (mg/y); *SD* standard deviation, *SF* serum ferritin, *TS* transferrin saturation

^b^ Mean TS of A*03 homozygotes was greater than that of A*03 heterozygotes (*p* < 0.05, post-hoc Scheffe's test)

^c^ Median SF of A*03 homozygotes was greater than that of A*03 heterozygotes (*p* < 0.05, post-hoc Dunn's test)

^d^ Testing was indicated in 61 men, 6 of whom had hypogonadotropic hypogonadism

^e^ Median APRI of A*03 homozygotes was greater than that of A*03 heterozygotes (*p* < 0.05, post-hoc Dunn's test). Median APRI of A*03-negative probands was greater than that of A*03 heterozygotes (*p* < 0.05, post-hoc Dunn's test)

FIB-4 indices > 1.10 were observed in 47.6% of A*03 homozygous men, 47.0% of A*03 heterozygous men, and 64.7% of A*03-negative men. Percentages of men with FIB-4 > 1.10 did not differ significantly across A*03 subgroups (*p* = 0.4147). Median FIB-4 did not differ significantly across A*03 subgroups (Table 1).

APRI > 0.44 was observed in 47.6% of A*03 homozygous men, 22.7% of A*03 heterozygous men, and 52.9% of A*03-negative men. These percentages differed significantly across A*03 subgroups (*p* = 0.0160). Percentages of men with A*03 homozygosity and A*03-negative men with APRI > 0.44 were significantly greater than the percentage of men with A*03 heterozygosity with APRI > 0.44 (*p* = 0.0281 and 0.0143, respectively). Median APRI was significantly lower in A*03 heterozygous men than in A*03 homozygous men and A*03-negative men (Table 1).

### Phenotypes of 76 women with hemochromatosis

These results are displayed in Table 2. There were no significant differences in phenotypes of women with HLA-A*03 homozygosity, A*03 heterozygosity, or no A*03.

**Table 2:** Phenotypes of 76 women with hemochromatosis and *HFE* p.C282Y homozygosity ^a^

Phenotype	HLA-A*03 homozygotes, % (*n* = 16)	HLA-A*03 heterozygotes (*n* = 38)	No HLA-A*03, % (*n* = 22)	Value of *p*
Mean age, y ± 1 SD	49 ± 15	51 ± 12	51 ± 13	0.8227
Mean TS, % ± 1 SD	77 ± 17	75 ± 20	76 ± 19	0.7844
Median SF, µg/L (range)	480 (65, 3468)	444 (34, 5427)	644 (32, 5000)	0.6635
Hemochromatosis arthropathy, % (n)	25.0 (4)	13.2 (5)	22.7 (5)	0.6063
Diabetes, % (n)	18.8 (3)	15.8 (6)	18.2 (4)	0.9357
Hypogonadotropic hypogonadism ^b^, % (n)	0	0	0	1.0000
Cardiomyopathy, % (n)	0	0	0	1.0000
Cirrhosis, % (n)	6.3 (1)	2.6 (1)	1.3 (1)	0.3030
Median FIB-4 index (range)	0.96 (0.52, 4.52)	0.93 (0.43, 5.16)	0.98 (0.44, 2.53)	0.8785
Median APRI (range)	0.22 (0.09, 1.47)	0.24 (0.13, 1.07)	0.24 (0.11, 1.17)	0.9252
Median QFe/age, mg/y (range)	77 (27, 291)	51 (7, 645)	54 (0, 244)	0.4346

^a^ Abbreviations: *APRI* aspartate aminotransferase-to-platelet ratio, *FIB-4* fibrosis-four variables index, *HLA* human leukocyte antigen, QFe/age is defined as the quotient of quantity of iron removed to achieve iron depletion by age at diagnosis (mg/y); *SD* standard deviation, *SF* serum ferritin, *TS* transferrin saturation

^b^ Testing was indicated in 3 women, none of whom had hypogonadotropic hypogonadism

FIB-4 indices > 1.10 were observed in 25.0% of A*03 homozygous women, 36.8% of A*03 heterozygous women, and 45.5% of A*03-negative women. These percentages did not differ significantly across A*03 subgroups (*p* = 0.4348). Median FIB-4 did not differ significantly across A*03 subgroups.

APRI > 0.44 was observed in 12.5% of A*03 homozygous women, 15.8% of A*03 heterozygous women, and 18.2% of A*03-negative women. These percentages did not differ significantly across A*03 subgroups (*p* = 0.8936). Median APRI did not differ significantly across A*03 subgroups.

### QFe/age grouped by quartiles

These results are displayed in Table 3. Median QFe/age was significantly higher in men and women in the highest QFe/age quartile than men and women with QFe/age in the lowest three quartiles, respectively. Prevalence of HLA-A*03 positivity or A*03 allele frequency did not differ significantly in either men or women according to QFe/age quartile subgroup, respectively.

**Table 3:** QFe/age in hemochromatosis probands with *HFE* p.C282Y homozygosity ^a^

		**Highest quartile, QFe/age, mg/y (*n*** **= 26)**	**Lowest three quartiles, QFe/age, mg/y (*n*** **= 78)**	**Value of** ***p***
Men (*n* = 104)	Median QFe/age, mg/y (range)	1913 (1226, 7143)^b^	486 (0, 1217)^b^	< 0.0001
	HLA-A^a^03 positivity ^c^, % (n)	76.9 (20)	85.9 (67)	0.3583
	HLA-A^a^03 allele frequency ^d^ (n)	0.4615 (24/52)	0.5385 (84/156)	0.8535
		**Highest quartile, QFe/age, mg/y (*n*** **= 19)**	**Lowest three quartiles, QFe/age, mg/y (*n*** **= 57)**	**Value of ** ***p***
Women (*n* = 76)	Median QFe/age, mg/y (range)	1667 (1212, 6452)	444 (0, 1133)	< 0.0001
	HLA-A^a^03 positivity ^c^, % (n)	68.4 (13)	71.9 (41)	0.7765
	HLA-A^a^03 allele frequency ^d^ (n)	0.4737 (18/38)	0.4561 (52/114)	0.3424

^a^ Abbreviations: *HLA* human leukocyte antigen, QFe/age is defined as the quotient of quantity of iron removed to achieve iron depletion by age at diagnosis (mg/y)

^b^ These values did not differ significantly from corresponding values in women

^c^ Positivity is defined as either A*03 homozygosity or heterozygosity

^d^ Allele frequency is defined as the quotient of total A*03 alleles by total A alleles

### HLA-A*03 in present and previous hemochromatosis cohorts

HLA-A*03 positivity and allele frequency were highest in the present cohort (Table 4).

**Table 4:** HLA-A*03 in present and previous hemochromatosis cohorts ^a^

Region (n)	A*03 positivity, %^b^	A*03 allele frequency ^c^	Reference
Australia (129)^d^	39.5	0.2519	[13]
Alabama (43)^d^	58.1	0.3953	[14]
Italy (47)^d^	61.7	0.3936	[15]
Alabama (180)	78.3	0.4944	present study
Value of *p*	< 0.0001	< 0.0001	

^a^ Abbreviation: *HLA* human leukocyte antigen

^b^ Positivity is defined as either A*03 homozygosity or heterozygosity. Positivity in the Australia cohort was lower than that in the previous Alabama (*n* = 43), Italy, and the present Alabama (*n* = 180) cohorts (*p* = 0.0098, 0.0015, and < 0.0001, respectively; post-hoc Fisher's exact tests). Positivity in the previous Alabama (*n* = 43) and Italy cohorts was lower than that in the present Alabama (*n* = 180) cohort (*p* = 0.0106 and 0.0239, respectively; post-hoc Fisher's exact tests)

^c^ Allele frequency is defined as the quotient of total A*03 alleles by total A alleles. A*03 allele frequency in the Australia cohort was lower than that in the previous Alabama (*n* = 43) and present Alabama (*n* = 180) cohorts (*p* = 0.0133 and < 0.0001, respectively; post-hoc Fisher's exact tests). A*03 allele frequency was lower in the Italy cohort than in the present Alabama cohort (*p* = 0.0147; post-hoc Fisher's exact test)

^d^ Patients in these cohorts were not characterized by *HFE* genotyping

## Discussion

This study revealed no significant differences in prevalences of conditions related to iron overload at diagnosis and median QFe/age in referred men or women with *HFE* p.C282Y homozygosity, respectively, across HLA-A*03 homozygosity, A*03 heterozygosity, and no A*03 subgroups. A*03 positivity and allele frequency did not differ significantly in men and women grouped by the highest and lowest three quartiles of QFe/age. Consistent with the present findings, A*03 was not a significant modifier of survival in Alabama hemochromatosis probands with p.C282Y homozygosity [37]. Taken together, these observations demonstrate that A*03, the only known independent marker of the hemochromatosis AH [9, 13–15, 38], is not linked to a putative A*03-linked locus that significantly influences iron overload or related conditions in Alabama p.C282Y homozygotes, contrary to previous postulates [13–16].

Mean TS in the present men was significantly higher in those with HLA-A*03 homozygosity than those with A*03 heterozygosity but not those with no A*03. Mean TS in the present women did not differ significantly across HLA-A*03 homozygosity, A*03 heterozygosity, and no A*03 subgroups. Mean TS did not differ significantly in hemochromatosis probands grouped by A*03 in three previous studies [13–15]. In a genome-wide association study of hemochromatosis patients with *HFE* p.C282Y homozygosity, TS was significantly associated only with the rs3811647 polymorphism in intron 11 of the transferrin gene (*TF*, chromosome 3q22.1) [39].

Median SF in the present men with HLA-A*03 homozygosity was significantly higher than that of men with A*03 heterozygosity but not men with no A*03. Median SF in the present women did not differ significantly across HLA-A*03 homozygosity, A*03 heterozygosity, and no A*03 subgroups. Median SF was significantly higher in men with hemochromatosis and A*03 heterozygosity in one study [15], although SF was not significantly associated with A*03 in two other studies [13, 14]. In adults unselected for hemochromatosis, numerous non-iron factors influence SF levels [40].

The prevalence of hemochromatosis arthropathy in this study did not differ significantly in either men or women grouped by HLA-A*03 homozygosity, A*03 heterozygosity, and no A*03, consistent with two previous reports [14, 15]. Greater age and SF levels at diagnosis are associated with hemochromatosis arthropathy [41–43]. In contrast, hemochromatosis arthropathy is more prevalent and iron overload is less severe in p.C282Y homozygotes than in persons with non-*HFE* hemochromatosis [44]. A man with *HFE* p.C282Y homozygosity without iron overload had severe, progressive knee arthropathy typical of hemochromatosis [45]. Taken together, these observations suggest that non-iron factors are also important in the pathogenesis of hemochromatosis arthropathy in p.C282Y homozygotes [44, 46].

Diabetes in the present probands was classified exclusively as type 2 and its prevalence was not significantly different in either men or women grouped by HLA-A*03 homozygosity, A*03 heterozygosity, and no A*03, consistent with two previous reports [14, 15]. In another study of Alabama referred *HFE* p.C282Y homozygotes, type 2 diabetes was not associated with common HLA types and haplotypes [47]. A consistent association of HLA region genes with type 2 diabetes in subjects not selected for hemochromatosis was not identified in genome-wide association studies [48, 49]. Like the *HFE* gene [17], loci that are risk factors for type 1 diabetes, defined as autoimmune β-cell destruction and usually leading to absolute insulin deficiency [24], occur in or are linked to the major histocompatibility complex (chromosome 6p21.33) [50, 51], although diverse autoimmune conditions in 236 referred hemochromatosis probands with p.C282Y homozygosity did not include type 1 diabetes [52].

The overall prevalence of hypogonadotropic hypogonadism in men in this study was 5.8%. The prevalence of hypogonadotropic hypogonadism did not differ significantly in men grouped by HLA-A*03 homozygosity, A*03 heterozygosity, and no A*03, consistent with a previous report [15]. In another study of referred patients with hemochromatosis (87% *HFE* p.C282Y homozygotes), 6.4% of men had hypogonadotropic hypogonadism [25]. Hypogonadotropic hypogonadism was not diagnosed in any of the present women. In another study, 5.2% of women with hemochromatosis had hypogonadotropic hypogonadism [25].

Cardiomyopathy due to cardiac siderosis, not diagnosed in the present probands, occurred in 2.3–14.9% of adult hemochromatosis patients but was not associated with HLA-A*03 in two previous studies [14, 15]. Hemochromatosis cardiomyopathy occurs more often in patients with juvenile-onset hemochromatosis due to homozygosity or compound heterozygosity for pathogenic alleles in the gene that encodes hemojuvelin (*HJV,* chromosome 1q21.1) [1, 53].

The prevalence of cirrhosis in this study did not differ significantly in either men or women grouped by HLA-A*03 homozygosity, A*03 heterozygosity, and no A*03. Proportions of the present men or women with FIB-4 and APRI values above respective thresholds for increased severe hepatic fibrosis risk and median FIB-4 and APRI values across A*03 subgroups revealed no consistent positive association with A*03. In previous studies, cirrhosis in patients with hemochromatosis was significantly associated with A*03 in one report [15] but not in another [14]. In *HFE* p.C282Y homozygotes, cirrhosis is positively associated with SF > 1000 µg/L at diagnosis [54, 55] and age at diagnosis, diabetes, amount of daily alcohol intake, and QFe [56].

Iron overload severity defined as QFe/age was not significantly associated with HLA-A*03 in the present study of *HFE* p.C282Y homozygotes. In three studies published before the discovery of *HFE*, age-adjusted quantities of iron removed to achieve iron depletion [14, 15] and mean hepatic iron index [13] were significantly greater in hemochromatosis patients with A*03 homozygosity.

Patients with hemochromatosis in three previous studies of HLA-A*03 [13–15] and some of the present probands underwent A*03 serotyping. The other present probands underwent DNA-based typing. A*03 is detected equally by serotyping and DNA-based typing. Thus, disparate typing reagents and techniques would not account for differences in A*03 frequencies across the present and three previous hemochromatosis cohorts [13–15].

HLA-A*03 allele frequency and positivity were higher in the present cohort than in three previous cohorts [13–15]. Proportions of patients with hemochromatosis who have A*03 vary across regions of Europe and derivative countries [11, 13–15, 22, 57]. Regardless, geographic differences would not account for the significantly greater A*03 positivity in the present Alabama cohort than in a previous Alabama hemochromatosis cohort from the same clinic [14]. In addition, ancestry reports of white Alabamians [58] and white Australians [59] are similar, although A*03 allele frequency was significantly lower in the previous Australia hemochromatosis cohort [13] than in a previous Alabama [14] and the present Alabama cohorts. Thus, geographic differences in A*03 positivity and allele frequency are an unlikely explanation for disparities observed in the present study and those reported in three previous studies [13–15].

We postulate that greater HLA-A*03 positivity and allele frequency in the present cohort of *HFE* p.C282Y homozygotes is due in part to the inclusion of patients with *HFE* genotypes other than p.C282Y homozygosity in the three previous hemochromatosis cohort studies published before the discovery of *HFE* [13–15]. Most adults with *HFE* hemochromatosis who are not p.C282Y homozygotes have *HFE* p.C282Y/p.H63D [17, 60], p.H63D/p.H63D [17, 61], or p.S65C [62], the third most common pathogenic *HFE* allele [18]. Whereas p.C282Y is often linked to A*03, p.H63D is often linked to HLA-A*29 [63–65]. p.S65C was linked to HLA-A*26 in Spanish subjects heterozygous for p.S65C [64] and to HLA-A*32 in an Alabama patient with hemochromatosis [66]. In addition, iron overload severity is greater, on the average, in patients with hemochromatosis and p.C282Y homozygosity than patients with hemochromatosis and p.C282Y/p.H63D [60], p.H63D/p.H63D [61], and p.S65C genotypes [62].

Proportions of patients diagnosed to have hemochromatosis who were subsequently demonstrated to be *HFE* p.C282Y homozygotes after the discovery of *HFE* in 1996 [17] were 52–96% in France (5 reports) [67], 88–100% in Australia (2 reports) [1], 59% in Alabama (1 report) [16], 33–69%% in Italy (2 reports) [1], and a global total of 78% (17 reports) [67]. These observations support our postulate that respective Australia, Alabama, and Italy hemochromatosis cohorts published during the interval 1995–1996 before the discovery of *HFE* [13–15] included patients with *HFE* genotypes other than p.C282Y homozygosity.

The human major histocompatibility complex (chromosome 6p21.33) is inherited as a single haplotype of closely linked polymorphic genes, comprising Class I loci (including HLA-A, telomeric), Class II loci (centromeric), and Class III loci (including *TNF*) [68]. *TNF* encodes a proinflammatory cytokine (tumor necrosis factor-alpha, TNF-α) secreted predominantly by monocytes and macrophages. TNF-α released in vitro by blood monocytes from patients with hemochromatosis was significantly less than that of control subjects in two studies [69, 70], but not in a third similar study [71]. Quantities of iron removed by phlebotomy and hepatic iron indices in referred hemochromatosis patients with p.C282Y homozygosity were significantly higher in those with *TNF* -308G → A than in those homozygous for *TNF* -308G, after adjustment for other factors [72]. In contrast, allele frequencies of seven *TNF* polymorphisms in HEIRS Study participants with p.C282Y homozygosity grouped by high TS/SF and low TS/SF did not differ significantly [73]. Taken together, these observations neither confirm nor deny that either TNF-α or *TNF* alleles influence(s) iron metabolism significantly in patients with hemochromatosis.

Portuguese hemochromatosis patients with greater iron overload and lower blood CD8 + T-lymphocyte concentrations had a highly conserved ancestral 500 kb microhaplotype on chromosome 6p designated as A-A-T, whereas other patients with milder iron overload and higher blood CD8 + T-lymphocyte concentration had a highly conserved haplotype designated as G-G-G [74]. A subsequent study of patients with hemochromatosis and *HFE* p.C282Y homozygosity in Portugal, Norway, and Alabama did not confirm that A-A-T is associated with greater iron overload severity [75].

Strengths of this study include a large cohort of referred, unrelated adults with hemochromatosis, *HFE* p.C282Y homozygosity, and HLA-A typing and a database that includes complete observations on age, TS, SF, conditions related to overload, and QFe/age [36]. The present results do not exclude the possibility that a characteristic of *HFE* p.C282Y homozygotes not studied herein or a locus linked to pathogenic *HFE* alleles other than p.C282Y influences hemochromatosis phenotypes. It is unknown which *TNF* polymorphism(s) is typically linked to A*03 and whether the predominant *TNF* polymorphism-A*03 linkage is consistent in p.C282Y homozygotes who reside in different geographic regions. It was beyond the scope of this study to classify the severity of hemochromatosis arthropathy, to measure hepcidin levels, or to perform *TNF* allele typing.

## Conclusions

Our results suggest that an A*03-linked locus does not influence phenotypes in referred *HFE* p.C282Y homozygotes. It is unlikely that heritable factors that modify phenotypes of p.C282Y homozygotes are linked to the hemochromatosis ancestral haplotype.

## Data Availability

All supporting data of this article are included in the submitted manuscript or are available at Open Science Framework (https://osf.io/tq8x6/).

## Abbreviations

AH: Ancestral haplotype
ALT: Alanine aminotransferase
ANOVA: Analysis of variance
APRI: AST-to-platelet ratio index
AST: Aspartate aminotransferase
FIB-4: Fibrosis-four variables index
FSH: Follicle-stimulating hormone
HEIRS Study: Hemochromatosis and Iron Overload Screening Study
HLA: Human leukocyte antigen
LH: Luteinizing hormone
QFe: Quantity of iron removed to achieve iron depletion
QFE/age: Age-adjusted quantity of iron removed to achieve iron depletion
SD: Standard deviation
SF: Serum ferritin
TNF-α: Tumor necrosis factor-alpha
TS: Transferrin saturation
